# Systematic assessment of microneedle injection into the mouse cornea

**DOI:** 10.1186/2047-783X-17-19

**Published:** 2012-06-20

**Authors:** Mario Matthaei, Huan Meng, Imran Bhutto, Qingguo Xu, Edwin Boelke, Justin Hanes, Albert S Jun

**Affiliations:** 1 The Wilmer Eye Institute, Johns Hopkins Medical Institutions, 400 N Broadway, Baltimore, MD 21231, USA; 2 Department of Ophthalmology, University Medical Center Hamburg-Eppendorf, Martinistrasse 52, Hamburg 20246, Germany; 3 The Center for Nanomedicine, Johns Hopkins University, 400 N Broadway, Baltimore, MD 21231, USA; 4 Department of Radiology and Radiooncology, University Medical Center Duesseldorf, Moorenstrasse 5, Duesseldorf 40225, Germany

**Keywords:** Cornea: gene therapy, Intrastromal injection

## Abstract

**Background:**

Corneal intrastromal injection is an important mode of gene-vector application to subepithelial layers. In a mouse model, this procedure is substantially complicated by the reduced corneal dimensions. Furthermore, it may be difficult to estimate the corneal area reached by the volume of a single injection. This study aimed to investigate intrastromal injections into the mouse cornea using different microneedles and to quantify the effect of injecting varying volumes. A reproducible injection technique is described.

**Methods:**

Forty eyes of 20 129 Sv/J mice were tested. India ink was intrastromally injected using 30° beveled 33 G needles, tri-surface 25° beveled 35 G needles, or hand-pulled and 25° beveled glass needles. Each eye received a single injection of a volume of 1 or 2 μL. Corneoscleral buttons were fixed and flat mounted for computer-assisted quantification of the affected corneal area. Histological assessment was performed to investigate the intrastromal location of the injected dye.

**Results:**

A mean corneal area of 5.0 ±1.4 mm^2^ (mean ± SD) and 7.7 ±1.4 mm^2^ was covered by intrastromal injections of 1 and 2 μL, respectively. The mean percentage of total corneal area reached ranged from 39% to 53% for 1 μL injections, and from 65% to 81% for 2 μL injections. Injections using the 33 G needles tended to provide the highest distribution area. Perforation rates were 8% for 30° beveled 33 G needles and 44% for tri-surface beveled 35 G needles. No perforation was observed with glass needle; however, intrastromal breakage of needle tips was noted in 25% of these cases.

**Conclusions:**

Intracorneal injection using a 30° beveled 33 G needle was safe and effective. The use of tri-surface beveled 35 G needles substantially increased the number of corneal perforations. Glass needles may break inside the corneal stroma. Injections of 1 μL and 2 μL resulted in an overall mean of 49% and 73% respectively of total corneal area involved.

## Background

Effective corneal gene therapy will require safe and effective gene delivery. However, lacrimation, mucus, blinking of the eyelid, and tight junctions of the corneal epithelium are a significant obstacle to genetic vectors [1,2]. Accordingly, intrastromal or intracameral injections are generally used for delivery of genetic therapeutics to subepithelial corneal layers [1].

Corneal thickness substantially varies among different animal species [3]. It is reported to be approximately 354 μm in rabbits and 170 μm in rats, while it is only 89.2 to 123.8 μm in mice [3,4]. Therefore, many studies in animals using corneal injections have used species other than mouse. However, the basic advantages of a mouse model are well-known: genetic models are often already available or easily practicable, experimental methods are usually better established, and expenses are comparably low. Thus, some studies describing the technique of intrastromal injections in mice have been conducted [5-17]. According to the method first described by Epstein and Stulting [9], most of these studies used a 33 gauge (G) needle with a bevel of 30° or 45° and the injected volume generally varied up to 2 μL [5-7,9-13]. It is important to consider that this variation may cause differences in the corneal area reached by the injected fluid or therapeutic agent.

The major difficulties of injecting a mouse cornea are posed by its reduced thickness and the handling of a comparably large needle. Improved injection systems, like foot-pedal driven pumps and smaller gauged needles made of glass, are supposed to simplify the procedure but may cause problems like plugging or breakage of the needle tip, and they are more expensive than standard hand-held syringes [8,15].

The present article describes a modified and reproducible injection method using hand-held syringes. In a systematic assessment, we compared injections into the mouse cornea by different types of microneedles and syringes and quantified the area reached by injections of different volumes.

## Methods

### Animals

129 Sv/J mice were bought from the Jackson Laboratory (The Jackson Laboratory, Bar Harbor, ME, USA). Animals ranged in age from 6 to 8 months. All procedures occurred according to the Association for Research in Vision and Ophthalmology statement for the Use of Animals in Ophthalmic and Vision Research.

### Needles and syringes

**Figure 1:**
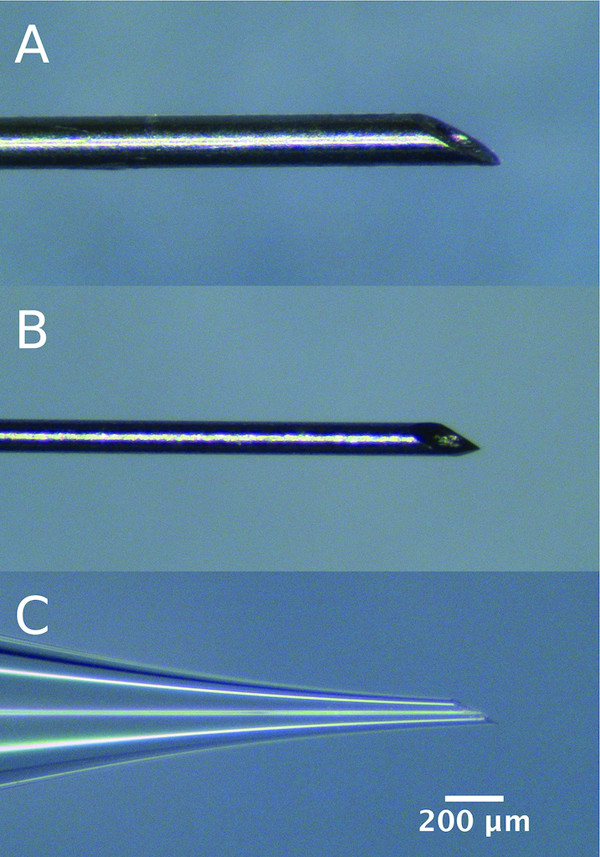
**Needles used for intrastromal injections. (A)** Hamilton 33 G 30° beveled, **(B)** WPI 35 G 25° tri-surface beveled, **(C)** WPI glass needle hand-pulled from 1 mm diameter glass capillary to an inner diameter of approximately 50 μm and 25° beveled.

Needles and syringes were obtained as follows (see Table 1 and Figure 1): 33 G needles (Hamilton, Reno, NV, USA) were attached to a 2.5 μL syringe (Hamilton); 35 G needles (WPI, Sarasota, FL, USA) were attached to a 10 μL syringe (WPI); glass needles were hand-pulled from 1 mm diameter glass capillaries (WPI) and beveled to 25° and an inner tip diameter of approximately 50 μm. Glass needles were attached to a 2.5 μL syringe (Hamilton) by a dual ferrule adaptor (Hamilton).

**Table 1:** Specification of microneedles used for intrastromal injections

**Needle type**	**Material**	**Bevel (degree)**	**Nominal outer diameter (mm)**	**Nominal inner diameter (mm)**	**Total length (mm)**
Hamilton, 33 G, point style 4	stainless steel	30	0.210	0.110	9.520
WPI, 35 G, tri-surface bevel	stainless steel	25	0.135	0.055	35.000
WPI, hand-pulled and beveled glass needle	glass	25	0.060^a^	0.050^a^	10.000^a^

^a^approximate measurements.

### Injected solution

Higgins Black India Ink (Chartpak, Leeds, MA. USA) was centrifuged for 10 min at 10,000 rpm. The supernatant was passed through a 0.2 μm sterile filter (Corning, Corning, NY, USA) prior to injection.

### Intracorneal injection technique

**Figure 2:**
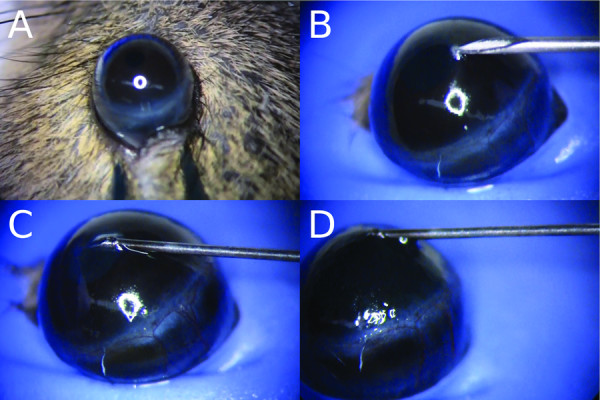
**Intrastromal injection of a mouse cornea. (A)** Proptosis of the eye by careful retraction of upper and lower lid with a forceps, **(B)** creation of a small intrastromal pocket in the mid-peripheral cornea using a 30 G needle, **(C)** insertion of a smaller needle and intrastromal advancement towards the corneal center, **(D)** forceful injection of India ink.

For anesthesia, a weight-adjusted combination of ketamine (100 mg/kg, Sigma-Aldrich, St. Louis, MO, USA), xylazine (20 mg/kg, Bioniche Pharma, Lake Forest, IL, USA) and acepromazine (3 mg/kg, Sigma-Aldrich) was administered by intraperitoneal injection. Both eyes received an intracorneal injection of dye. All injections were performed by the same investigator (MM) after a period of 1 to 2 months of weekly training. Before each injection, proparacaine hydrochloride 0.5% eye drops (Bausch and Lomb, Madison, NJ, USA) and 0.5% moxifloxacin eye drops (Vigamox; Alcon Laboratories, Fort Worth, TX, USA) were applied. All procedures occurred under a Nikon SMZ800 dissection microscope (Nikon, Melville, NY, USA). The anesthetized mouse was placed on a Styrofoam platform, and the head was stabilized using a thin strip of non-latex material fashioned from a disposable laboratory glove. The strip was thin enough not to occlude the nostrils of the mouse. A small hole was pre-cut into the strip to allow protrusion of the globe. The eye was cautiously proptosed by forceps retracting the upper and lower lid (Figure 2A). After fixation under the strip, the proptosis was maintained by tension along the margins of the pre-cut hole and the forceps were withdrawn. Care was taken that the strip did not strangle the central ocular blood vessels and optic nerve.

In the corneal mid-periphery, a small nick was made down to the anterior stroma with a 30 G beveled needle (Becton Dickinson, Franklin Lakes, NJ, USA) (Figure 2B); the needle was slightly moved tangentially and upwards without withdrawal to confirm stromal placement. This created a small intrastromal pocket by separating the stromal lamellae, as could be observed by opacification of the anterior stroma (Figure 2B). A second smaller needle (see Table 1) was inserted into the pocket (Figure 2C). It was slowly tangentially pushed forward towards the corneal center until the tip of the needle had reached a position where approximately 0.5 mm of the needle shaft had entered the corneal stroma (Figure 2C). At this point, the India ink was forcefully injected without moving the needle tip (Figure 2D), and the needle subsequently was retracted. A perforation of the cornea was indicated by a sudden flattening of the anterior chamber, a temporary darkening of the aqueous humor by the ink, or leakage of clear aqueous humor from the injection site. All eyes with a corneal perforation were excluded for subsequent investigations from the study. Mice were euthanized 30 minutes after the injection, and both eyes were enucleated for further assessment, as described below.

### Quantitative analysis

After enucleation, whole globes were fixed in 4% paraformaldehyde in phosphate-buffered saline for 1 h at room temperature and the tissue was equilibrated in 20% sucrose solution at 4°C overnight. Subsequently, corneal buttons, including a narrow scleral rim, were excised. Four radial incisions were made and corneas were flat mounted on a glass slide. Pictures were taken at 5× magnification under the dissection microscope using a Canon VIXIA HFS21 digital camera (Canon, Lake Success, NY, USA). The injected corneal area could clearly be identified by a black staining of the tissue. The borders of the total corneal area were marked by Schwalbe’s line. The injected area and total corneal area were measured using the ImageJ 1.43 software (National Institutes of Health, Bethesda, MD, USA).

### Histological evaluation

Eight mouse eyes were processed for histological evaluation after intracorneal injection to assess the intrastromal location of the injected fluid. After enucleation, eyes were fixed as described above. Excised corneoscleral buttons were embedded in Tissue Tek OCT compound (Sakura Finetek, Torrance, CA, USA) at −80°C. Cryosections of 10 μm were examined by hematoxylin-eosin staining.

### Statistics

The stained areas of different groups were compared using the one-tailed Mann–Whitney nonparametric test. A *P*-value of <0.05 was regarded as statistically significant. Statistical software used included Excel 2008 for Mac (Microsoft, Redmond, WA, USA) and GraphPad Prism 4 (GraphPad, La Jolla, CA, USA).

## Results

**Figure 3:**
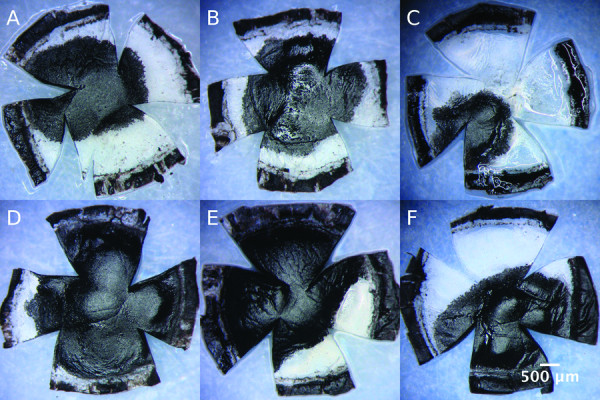
**Flat mounts of 4% paraformaldehyde-fixed mouse corneas after intrastromal India ink injections. (A,B,C)** Mouse corneas after injection of 1 μL and **(D,E,F)** 2 μL using a 33 G 30° beveled needle (A and D), a 35 G 25° tri-surface beveled needle (B and E) and a hand-pulled and beveled glass needle (C and F).

The mean total corneal endothelial surface was 10.52 ±0.74 mm^2^. Examples of flat mounted murine corneas after 1 and 2 μL injection using different needles are presented in Figure 3. Intracorneal injections of a volume of 1 μL India ink resulted in a significantly (*P*<0.05) smaller mean affected area (5.0 ±1.4 mm^2^) than injections of a volume of 2 μL (7.7 ±1.4 mm^2^). This difference was also significant (*P*<0.05) for 1 and 2 μL injections with each needle type.

**Figure 4:**
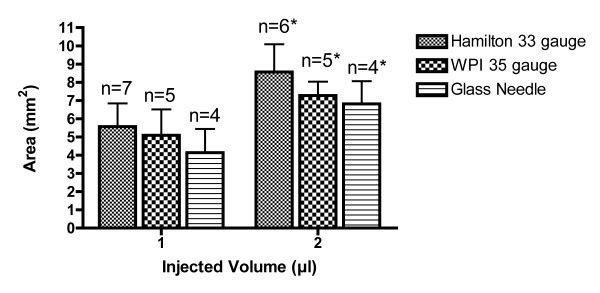
**Corneal area affected after intrastromal injection of 1 μL or 2 μL India ink using different needle types.** * *P*<0.05 compared with 1 μL injection using same needle type.

**Figure 5:**
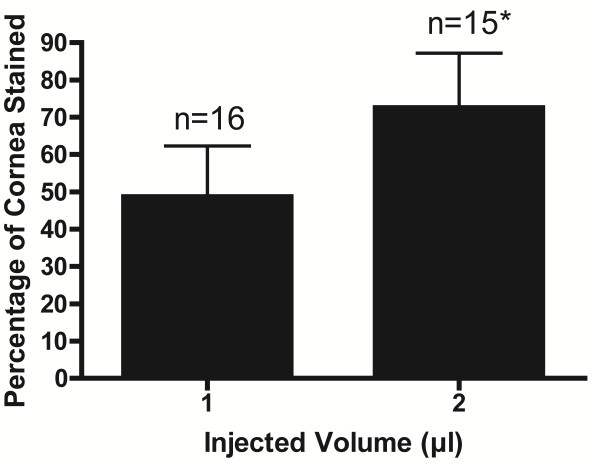
**Percentage of corneal area affected after intrastromal injection of 1 or 2 μL India ink.** * *P*<0.05 compared with 1 μL injection.

Injections using a Hamilton 33 G needle showed the tendency to affect the largest absolute dyed areas (1 μL: 5.6 ±1.3 mm^2^, 2 μL: 8.6 ±1.6 mm^2^), while injections with WPI 35 G needles (1 μL: 5.1 ±1.4 mm^2^, 2 μL: 7.3 ±0.8 mm^2^) and glass needles (1 μL: 4.1 ±1.3 mm^2^, 2 μL: 6.8 ±1.2 mm^2^) tended to provide smaller absolute affected areas (Figure 4). However, these differences between needles showed no statistical significance. The percentages of total corneal areas that were involved after a single injection of 1 or 2 μL ink are displayed for each needle type in Table 2. The mean proportionally affected corneal area was 49 ±14% for 1 μL volume of injection and 73 ±15% for 2 μL volume of injection, as displayed in Figure 5 (*P*<0.05).

**Table 2:** Percentage of total corneal area affected after intrastromal injection of India ink

**Needle type**	**1 μL injection**				**2 μL injection**			
	**Percentage of total area affected (mean ± SD)**			**n**	**Percentage of total area affected (mean ± SD)**			**n**
Hamilton, 33 G	53.28	±	12.37	7	80.63	±	14.28	6
WPI, 35 G	50.43	±	16.14	5	65.29	±	6.75	5
WPI, glass needle	38.67	±	9.03	4	69.65	±	19.09	4
Total	48.74	±	13.59	16	72.59	±	14.60	15

A perforation of the cornea occurred in one case (8%) using 33 G needles, in eight cases (44%) using 35 G needles, and in no case using hand-pulled glass needles. For the glass needles, a breakage of the tip occurred in two cases (25%). A small amount of leakage was noted in all cases.

**Figure 6:**
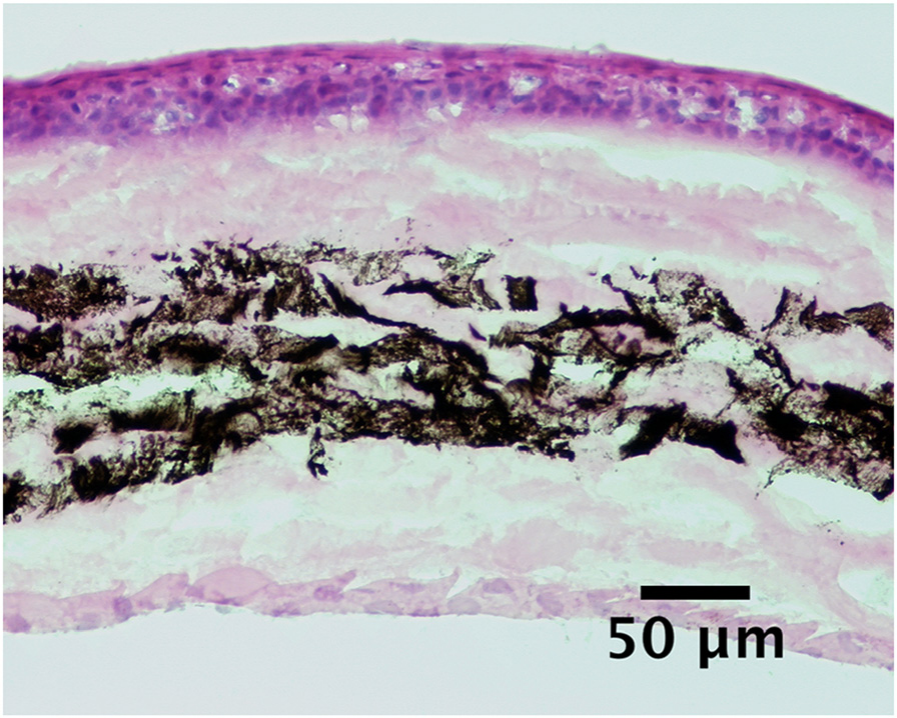
**Histology of a mouse cornea after intrastromal injection of India ink.** Separation of the stromal lamellae and intrastromal dispersion of the India ink. Hematoxylin and eosin staining.

Histological assessment of injected corneas revealed a separation of the stromal lamellae and an interlamellar dispersion of the India ink in all cases, as can be seen in Figure 6.

## Discussion

Due to its clarity, accessibility and clinical relevance, the cornea provides an excellent tissue for studies of gene transfer [18-20]. Gene delivery into subepithelial layers via topically-administered drops is hampered by tear flow, protective mucus layers, blinking and epithelial tight junctions. Intrastromal injection provides an important route to overcome these barriers. Despite the advantage of mouse models for corneal gene therapy studies, intrastromal injections in mice are difficult due to the small corneal thickness, and studies using intrastromal injections in the mouse cornea report corneal perforation rates up to 15% [9,15].

In mice, in contrast to humans, the corneal thickness increases towards the center [21]. This largely explains the method commonly used for intrastromal injection in mice, which involves a nick in the epithelium and anterior stroma made in the mid-periphery with a larger (30 to 32 G) needle. Subsequently, a finer needle is introduced and pushed forward, thereby separating the stromal fibers towards the center where the injection occurs. As described above in detail, we also applied this method but modified the mode of globe fixation.

The aim of this study was a systematic comparison of intrastromal injections using different microneedles in the mouse eye. Furthermore, our goal was to assess the corneal areas that may be affected by single injections of different volumes. Overall, the ratio between injected volume and the affected corneal area was reproducible. However, the ratio of injected volume and affected corneal area did not rise proportionally. This may have been caused by the leakage that was observed in all cases. The leakage is likely to rise with increasing volume of the injection, perhaps due to increased pressure inside the stroma. The volume of leakage was difficult to quantify, but appeared noticeably greater for the larger (2 μL) injection volumes. Regardless, injected fluid was delivered to a large portion of the corneal surface area with 2 μL injected volume.

There was a trend towards a reduced involved area with a decrease of the inner diameter of the microneedle. However, these results were not statistically significant.

The perforation rate of 8% that was achieved with the 33 G needle is acceptable for practical use in most experimental studies. Using the tri-surface beveled 35 G needle, a perforation rate as high as 44% was observed. This was most likely caused by the sharp tip of the tri-surfaced bevel that did not give rise to a separation of the stromal fibers, but rather caused an easier penetration of the anterior chamber. The best results with regard to the rate of perforation were achieved using glass needles. However, in 25% of all cases, a breakage of the glass needle tip occurred inside the corneal stroma. This may cause additional tissue trauma in the postoperative course, which may be undesirable.

It was previously reported that a major drawback of hand-held syringes for injection of mouse eyes may be the difficulty of keeping the needle tip in the injection area while depressing the plunger [15]. According to our experience, this problem is mainly caused by ineffective eye-fixation using forceps that are held with the non-injecting hand. The above-mentioned method of fixation with a strip of elastic material allowed for safe proptosis and fixation of the eye so that the injection could be administered more reliably. One disadvantage of this technique is the low risk of compression of the ophthalmic artery, vein or optic nerve, and care must be taken to avoid this complication by proper placement of the strip.

The use of a gas-powered or foot-pedal driven microinjection system may lead to a smoother course of the injection procedure and may, therefore, prevent the breakage of glass needle tips within the cornea. In this case, the use of hand-pulled glass needles could provide a safe and atraumatic method for intrastromal injections. When using hand-held syringes as in our study, however, the use of 33 G metal needles provided the most reliable and effective outcomes.

## Conclusions

Using the described technique intracorneal injection with a 30° beveled 33 G needle was safe and effective. The method presented here may be used as an easily reproducible guideline for intracorneal injections into the mouse cornea, and should facilitate use of mouse models for corneal gene therapy studies.

## Competing interests

The authors declare that they have no competing interests.

## Authors’ contributions

MM designed the study, developed the injection technique, performed the injections and analyses, and prepared the manuscript; HM and QX participated in the performance of analyses, study design, and preparation of manuscript; IB participated in the development of the injection technique and preparation of the manuscript; EB and JH contributed to the study design and preparation of the manuscript; ASJ designed the study, developed the injection technique and prepared the manuscript. All authors read and approved the final manuscript.
